# Complete mitochondrial DNA sequence of *Brachysomophis crocodilinus* (Anguilliformes: Ophichthidae)

**DOI:** 10.1080/23802359.2017.1307707

**Published:** 2017-03-29

**Authors:** Xiaojing Song, Wenqiao Tang

**Affiliations:** a Laboratory of Ichthyology, Shanghai Ocean University, Shanghai, China;; b Shanghai Key Laboratory of Marine Animal Taxonomy and Evolution, Shanghai, China

**Keywords:** *Brachysomophis crocodilinus*, Ophichthidae, mitochondrial genome, phylogenetic analysis

## Abstract

*Brachysomophis crocodilinus* belongs to the family Ophichthidae, the complete mitochondrial genome of which was sequenced in this study. The mitochondrial genome of *B. crocodilinus* is of 17,818 bp in length, with overall base composition of 32.11% A, 24.69% T, 16.22% G, and 26.98% C. The genome content includes 13 protein-coding genes, 22 tRNA genes, 2 rRNA genes, and 2 control regions. The result of phylogenetic analysis indicates that *B. crocodilinus* mitogenome is close to that of *Ophisurus macrorhynchos*, which are nested within the family Ophichthidae.

Ophichthidae is a family of the order Anguilliformes, containing 59 genera and about 319 species. The distinguishing features of the family Ophichthidae are posterior nostril usually within or piercing upper lip; median supraorbital pore in frontal sensory canal; caudal fin absent; pectoral fins present or absent; vertebrae 110–270 (Nelson et al. 2016). *Brachysomophis crocodilinus* belongs to the family Ophichthidae, which distributes in the Indo-West Pacific; including East and South China Sea (Zhang 2010). In this study, the complete mitochondrial genome of *B. crocodilinus* was determined. Fish sample was collected from the Changjiang Estuary (30°51′16.24″N, 121°54′46.51″E), Shanghai, China. The specimen was stored in the Laboratory of Ichthyology, Shanghai Ocean University, with accession number 20160613. The sample DNA is available upon request.

Mitochondrial DNA is a maternally inherited circular genome that serves important functions in metabolism and population genetics (Boore 1999). In the present study, the sequenced mitochondrial genome of *B. crocodilinus* (GenBank accession number: KY081398) is of 17,818 bp in length, with overall base composition of 32.11% A, 24.69% T, 16.22% G, and 26.98% C. With the exception of two control regions, the genome content of *B. crocodilinus* includes 2 rRNA, 22 tRNA, and 13 protein-coding genes, as found in other vertebrates (Inoue et al. 2004). The mitogenome exhibits the translocation of *nad6* in some species of the order Anguilliformes (Shen et al. 2014). The phenomenon also occurs in *B. crocodilinus*, arrangement of protein-coding genes of which is: *nad1*—*nad2*—*cox1*—*cox2*—*atp8*—*atp6*—*cox3*—*nad3*—*nad4L*—*nad4*—*nad5*—*cob*—*nad6*. Apart from the *ND6* gene and eight tRNA genes (*tRNA-Gln*, *Ala*, *Asn*, *Cys*, *Tyr*, *Ser*, *Glu* and *Pro*) encoded on the L-strand, most genes are on the H-strand. 11 of 13 protein-coding genes start with a common initiation codon ATG, while *COI* and *ATP6* utilize GTG. According to the result given by Mitoannotator (Iwasaki et al. 2013), there are six protein-coding genes with the order of *ND2*, *COII*, *ATP6*, *COIII*, *ND3*, and *ND4* ending with incomplete stop codon (T––, T––, TA–, TA–, T––, T––), while six genes (*ND1*, *COI*, *ATP8*, *ND4L*, *ND5*, *Cytb*) use the stop codon TAA, and *ND6* uses the stop codon TAG. The end of *ATP8* overlaps with the beginning of *ATP6* with a length of 10 bp, and there is another overlap between *ND4L* and *ND4* with a length of 7 bp.

To investigate the phylogenetic relationship among the order Anguilliformes, we downloaded the mitochondrial genome sequences of 13 currently available species of Anguilliformes, including *Anguilla australis* (AP007235), *A. obscura* (AP007247), *Coloconger cadenati* (AP010863), *Facciolella oxyrhyncha* (AP010866), *Heteroconger hassi* (AP010859), *Hoplunnis punctata* (AP010865), *Ilyophis brunneus* (AP010848), *Myrichthys maculosus* (AP010862), *Nessorhamphus ingolfianus* (AP010850), *Nettastoma parviceps* (AP010864), *Ophichthus rotundus* (KY081397), *Ophisurus macrorhynchos* (AP002978) and *Paraconger notialis* (AP010860), together with African lungfish *Protopterus annectens* (NC018822) as an outgroup species. The phylogenetic tree was constructed using MEGA6 (Tamura et al. 2013) for neighbour-joining method. Tree topology was evaluated by 1000 bootstrap replicates. The result indicates that *B. crocodilinus* mitogenome is close to that of *O. macrorhynchos*, which are nested within the family Ophichthidae (Figure 1).

**Figure 1.**
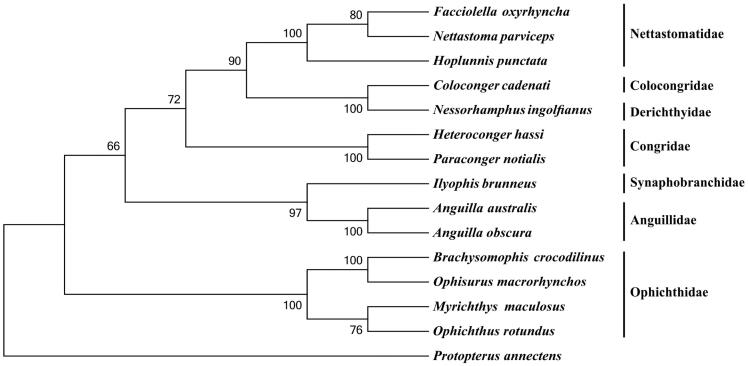
Phylogenetic tree of order Anguilliformes, with African lungfish *P. annectens* as an outgroup. The topology of phylogenetic tree was inferred from neighbour-joining method.
